# Multipoint Pacing with Fusion-optimized Cardiac Resynchronization Therapy: Using It All to Narrow QRS Duration

**DOI:** 10.19102/icrm.2021.120102

**Published:** 2021-01-15

**Authors:** Raffaele Corbisiero, Andrew Mathew, Caitlin Bickert, David Muller

**Affiliations:** ^1^Deborah Heart & Lung Center, Browns Mills, NJ, USA; ^2^Abbott Laboratories, Sylmar, CA, USA

**Keywords:** Cardiac resynchronization therapy, fusion optimization, multipoint pacing

## Abstract

Adaptive atrioventricular (AV)-shortening algorithms have achieved QRS duration (QRSd) narrowing in traditional cardiac resynchronization therapy (CRT) patients. Multipoint pacing (MPP) has also demonstrated benefit in this population. An additional site of activation via intrinsic conduction of the septum may further contribute to CRT; however, the incorporation of all strategies together has yet to be explored. We therefore developed and tested a method combining MPP-CRT and controlled septal contribution to create a multifuse pacing (MFP) technique, establishing four ventricular activation sites for CRT patients using measurements from intracardiac electrograms (EGMs) and incorporating an AV-delay shortening algorithm (SyncAV™; Abbott Laboratories, Chicago, IL, USA) to narrow the QRSd. Patients in sinus rhythm with an AV conduction time of less than 350 ms were included in this analysis and were further stratified by strictly defined left bundle branch block (sLBBB) or nonspecific intraventricular conduction delay (IVCD). EGM-based measurements to determine the QRS septal onset to right ventricular (RV) time (SRAT) and the left ventricular (LV) to RV pacing conduction time were collected and applied to a formula to facilitate MFP. QRSd was compared between before and after programming. A total of 22 patients (19 men and three women) with similar baseline characteristics were compared (all values in mean ± standard deviation). The overall baseline QRSd of 153.31 ± 24.60 ms was decreased to 115.31 ± 16.31 ms after MFP programming (p < 0.0001). The measured SRAT was 59.40 ± 28.49 ms, resulting in a negative AV offset of −20.0 ± 24.97 ms. Patients in the sLBBB group (n = 7) were aged 67.8 ± 13.3 years and had a QRSd of 168.85 ± 27.29 ms that decreased to 113 ± 16.69 ms for a reduction of 55.42 ± 19.3 ms or 32.1% (p = 0.0003). In the IVCD group (n = 15), the baseline QRSd of 146.06 ± 20.29 ms was decreased to 116 ± 16.66 ms for a reduction of 30.07 ± 16.41 ms or 20.62% (p = 0.0001). When comparing the sLBBB and IVCD groups, the sLBBB group was favored by a reduction of 25.35 ms (p = 0.00046). Ultimately, MFP achieved statistically significant reductions in QRSd in all patients tested in this analysis. The benefit was also significantly better in the sLBBB group as compared with in the IVCD group.

## Introduction

Cardiac resynchronization therapy (CRT) is a well- established treatment for patients with symptomatic systolic heart failure (HF) and interventricular conduction abnormalities.^1–4^ Although this therapy has shown great benefit in numerous clinical trials, nonresponse remains substantial, ranging from 25% to 30% depending on how response is defined.^5,6^ In screening for CRT, concentration and attention to the QRS duration (QRSd) are critical to ensure that adequate prolongation exists in association with the New York Heart Association (NYHA) class to meet current guidelines.^7^ Additionally, when the morphology in the strict definition of left bundle branch block (sLBBB) includes left ventricular (LV) conduction delay evidenced by a QRSd of at least 140 ms in men or at least 130 ms in women. Meanwhile, the adoption of mid-QRS notching in at least two of the leads I, aVL, V1, V2, V5, and/or V6 has shown clear benefit when using the sLBBB criteria over incomplete or generalized intraventricular conduction delay (IVCD) when CRT therapy is initiated.^8–10^

Some studies have suggested that one-third of patients diagnosed with LBBB may not have complete LBBB but instead an incomplete bundle with some degree of LV enlargement; interestingly, this patient group is of a similar size to the CRT nonresponder population.^11–14^ However, the addition of multiple sites of LV activation with CRT [multipoint pacing (MPP)] was not available at the time of most previous studies and may have led to different outcomes.

The use of reduction in QRSd in the determination of long-term clinical CRT response is inconsistent; however, most data support this method and meta-analysis shows favorable outcomes when using QRS narrowing as a surrogate for optimal CRT delivery.^15–18^

Recent work by Varma et al. discussed the use of an adaptive atrioventricular (AV) algorithm (SyncAV™; Abbott Laboratories, Chicago, IL, USA) demonstrating superiority over conventional out-of-the-box settings, LV-only, and static AV delays in the narrowing of QRSd with bipolar LV programmed devices.^19^ New adaptations to LV pacing have also provided the ability of an additional wavefront in the form of MPP from two poles on a quadripolar LV lead. These studies have not only reduced the rate of nonresponse but equally have decreased the QRSd.^20–23^ In the Italian Registry on Multipoint LV Pacing (IRON-MPP) trial, when MPP therapy was added, an adjunctive significant reduction in QRSd was noted (p < 0.001) and was optimized 38% according to the configuration providing the narrowest QRSd, with a final mean reduction of 134.8 ms versus that of 141.3 ms achieved with conventional biventricular pacing.^24^

CRT optimized to provide some intrinsic fusion has also been shown to demonstrate significant reductions in QRSd.^25,26^ The addition of septal activation to contribute still further to the narrowing of QRSd may theoretically provide additional benefit. Interestingly, combining the use of an adaptive AV algorithm with the concomitant use of MPP is currently being evaluated; however, optimizing these features with the addition of fusion in a standardized manner has yet to be examined.

We developed a novel series of device-based measurements, which were verified on surface electrocardiograms (ECGs), to suggest a patient-specific usage of all these methods concomitantly. In addition to its use at the time of implant, this method is easily able to be repeated at subsequent follow-up visits.

## Methods

As all the techniques and algorithms in this analysis are approved by the United States Food and Drug Administration (FDA) and the programming is temporary in nature, our institutional review board waived the need for a formal protocol submission. Our analysis consisted of 22 consecutive patients (19 men) selected at implant based on American College of Cardiology/American Heart Association guidelines. Patients in sinus rhythm with intact AV conduction, a model 1458Q Quartet quadripolar LV lead (Abbott Laboratories), and a device-based delay of less than 350 ms were included in this analysis due to the programming limitations of Abbott Laboratories’ CRT defibrillator (CRT-D) delays. Patients were further stratified by sLBBB (n = 7) as defined earlier or nonspecific IVCD (n = 15) and excluded patients with right BBB (RBBB) as they are not currently CRT indicated. The measurements were performed via the Merlin™ 3650 (Abbott Laboratories) using screen calipers to determine conduction times and validated with surface ECG findings to guide programming. QRS duration was measured in a 50-mm sweep and encompassed from when the QRS left the baseline until it returned to the baseline. Measurements were performed by a single investigator to eliminate potential variability. All patients were programmed using the criteria described below and then each result was compared with their baseline QRS value.

### Intrinsic contribution

**Figure 1:**
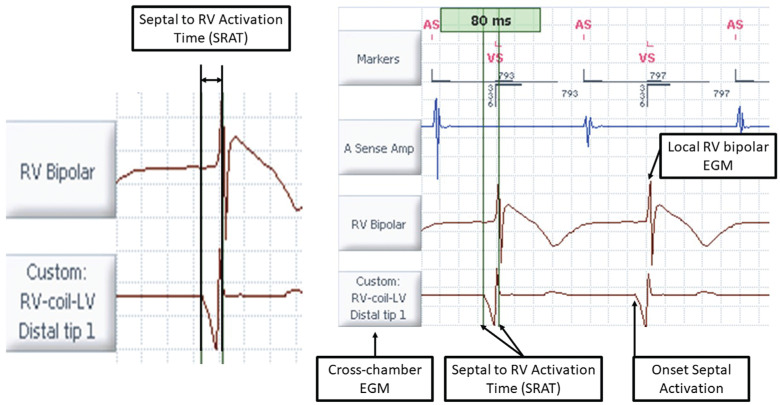
Intracardiac EGM measurements to determine settings and measurement of the SRAT (left). The distance between the RV coil and LV distal tip was used to visualize the onset of septal activation and is measured to the positive peak of the RV bipolar EGM. This confirms the SRAT in this example to be 80 ms. EGM: electrogram; LV: left ventricular; RV: right ventricular; SRAT: septal onset to right ventricular time.

All QRS measurements were made in lead I on the Merlin™ 3650 programmer (Abbott Laboratories) for standardization and to ensure uniformity of the wavefront deflections and were confirmed on the electrophysiology (EP) monitoring system at implant consisting of leads I, II, III, aVL, aVR, and V1. Lead aVR was used on the programmer during LV pacing for comparison of the RV coil–to–LV tip electrograms (EGMs). All QRSd measurements were made from the earliest deflection from baseline as the QRS onset until the latest activation returned to the baseline. Intracardiac measurements for the intrinsic contribution were calculated and ensured by measuring the onset of septal activation, which was determined using a cross-chamber EGM vector (with the RV coil as the cathode and LV tip as the anode). Septal onset was considered to have occurred when the EGM left baseline. On-screen calipers were used to measure the septal onset to the first deflection on the RV bipolar channel to determine the septum to RV activation time (SRAT) **(Figure 1)**.

### LV pacing measures

**Figure 2:**
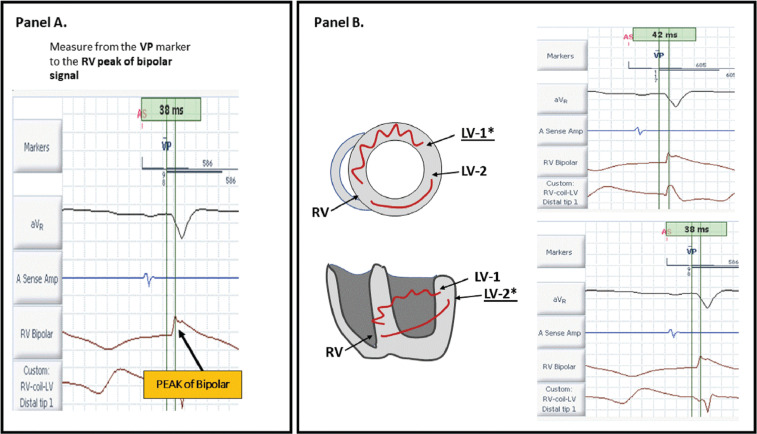
Determining the MPP sequence. **A:** The VP marker indicates the onset of measurement and is measured to the peak of the bipolar EGM to determine the LV–RV conduction time. **B:** As LV-1* is stimulated (top), the time from LV-1 (VP marker) until the RV bipolar is 42 ms. When LV-2* is stimulated (bottom), the LV-2 (VP marker) until the RV bipolar time is 38 ms. In this instance, LV-1 would be the first MPP programmed vector, followed by LV-2, as the LV–RV time is longer in LV-1 than in LV-2. EGM: electrogram; LV: left ventricular; MPP: multipoint pacing: RV: right ventricular; VP: ventricular pacing.

Capture, threshold, and absence of phrenic nerve stimulation were tested on all poles. The two vectors with lead–electrode separation of greater than 30 mm and the best capture measurements along with the absence of phrenic stimulation were selected to provide the optimal MPP programming as per our standard practice. Each of the two vectors was separately programmed to an LV-only mode, a screen capture was taken, and calipers were used to determine the LV-pacing to RV-bipolar time to establish the LV–RV activation time. This was repeated for the second selected vector **(Figure 2)**.

### Programming

To provide MPP therapy with septal and RV pacing activation and simultaneously develop multifuse pacing (MFP) programming, the SRAT *0.5 dictated the SyncAV offset. Next, the longer of the two LV–RV times was programmed as the LV-1 contributor in the programming sequence, while the shorter LV–RV time was programmed as the LV-2 contributor. This allows for wavefronts from the septum, LV-1, LV-2, and the RV, all of which should be programmed to as simultaneous an offset as possible, to each contribute to activation. Fusion was confirmed by reducing each ventricular output individually to confirm minute changes in QRS morphology.

### Statistical analysis

Following successful CRT implantation in a series of consecutive patients, MFP programming methods were implemented. Data were analyzed using Microsoft Excel (Microsoft Corporation, Redmond, WA, USA). Continuous data are presented as mean ± standard deviation. Matched pair and independent group t-tests between means were performed using StatPac for Windows (StatPac, Inc., Northfield, MN, USA), with the p-value considered significant at less than 0.05.

## Results

The group consisted of 22 patients (19 men and three women) with an age of 66.63 ± 9.71 years, all in sinus rhythm. The baseline characteristics can be seen in **Table 1**. The overall group’s mean baseline QRSd was 153.31 ± 24.60 ms, which decreased to 115.31 ± 16.31 ms after MFP use (p < 0.0001). The mean measured SRAT was 59.40 ± 28.49 ms, resulting in a mean SyncAV offset of 20.0 ± 24.97 ms.

The sLBBB group (n = 7) had a mean age of 67.8 ± 13.3 years with a mean QRSd of 168.85 ± 27.29 ms. The mean SRAT was 47.14 ± 28.85 ms and the programmed SyncAV offset was 17.14 ± 21.38 ms. The longest LV pace to RV sense period was 116 ± 70.32 ms and the shortest was 74.83 ± 50.8 ms. Use of MFP in this group resulted in a final QRSd of 113 ± 16.69 ms, for a reduction of 55.42 ± 19.3 ms or 32.1% (p = 0.0003).

In the IVCD group (n = 15), the mean age was 66.06 ± 8.0 years, with a mean baseline QRSd of 146.06 ± 20.29 ms. The mean SRAT was 65.1 ± 27.3 ms and the programmed SyncAV value was 21.3 ± 27.1 ms. The longest LV pace to RV sense time was 139 ± 62.08 ms and the shortest was 115.7 ± 58.0 ms. Use of the MFP resulted in a final QRSd of 116 ± 16.66 ms, for a reduction of 30.07 ± 16.41 ms or 20.62% (p = 0.0001) **(Table 2)**.

**Figure 3:**
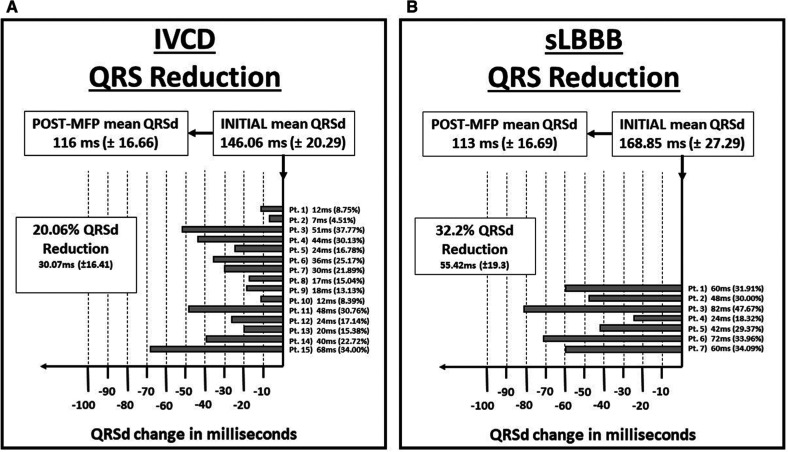
Changes in QRS duration between groups based on baseline ECG criteria. ECG: electrogram; QRSd: QRS duration; MFP: multifuse pacing**. A:** Patients with generalized IVCD pattern. IVCD: intra-ventricular conduction delay. **B:** Patients with strict LBBB criteria. LBBB: left bundle branch block.

When comparing the sLBBB and IVCD groups, the mean reduction favored the sLBBB group by 25.35 ms (p = 0.00046) **(Figure 3)**.

## Discussion

This analysis highlighted a few interesting points. First, while using the sLBBB criteria, we observed a significantly longer baseline QRSd as compared with in the general IVCD population. One would think this native intrinsic delay would equate to a longer LV to RV time as well; however, the inverse was actually noted, as the mean longest LV to RV time was 23 ms longer in the IVCD group. However, even given that the IVCD group had an extended LV to RV time, there was still a greater percentage of reduction in the sLBBB group. In theory, this may be attributed to the addition of the septal contribution occurring at the same time as that of the latest LV–RV site and its overall effects on QRSd. In addition to this point, the question of whether there is a contribution of RV pacing at all in patients receiving CRT has come under scrutiny as it alters the activation of both ventricles,^25^ particularly at the apex and septal wall. This may also explain why LV-only (> 80%) pacing, when combined with native activation like in the AdaptResponse clinical trial, seemed more beneficial than traditional CRT which includes RV pacing. In our method, the potential deleterious effects of RV pacing are felt to be decreased as multiple wavefronts are initiated.^26^

Although our study lacked a control group of MPP programming without fusion, the final QRSd values in our analysis were substantially lower than those reported in the IRON-MPP trial, even despite that the majority of programming in that study was tailored to the narrowest QRSd (115.31 vs. 134.8 ms).^24^

Similar effects have been demonstrated in prior studies in which tailoring the programming of the AV negative offset was generally determined by observation and intentional fusing of the QRS morphology, thus decreasing its duration on ECG.^19^ As the right atrial (RA) sense to RV sense was determined and used for the SyncAV measurement programming for 256 cycles, the offset delay is based on this RA–RV time delay. As such and with the AV node (AVN) having rich sympathetic innervation and decremental properties, which accounts for some portion of the RA–RV time, we had to identify the end of the AV nodal contribution and the beginning of the septal contribution. Much like using the His signal in an EP study to indicate the end of the AVN, this was a key component to determining the proper programming to ensure continual CRT delivery. As MFP uses the SRAT, which only accounts for ventricular contribution and is defined as the portion of time from septal to RV activation, the SRAT offset once determined allows the RV-pacing contribution to consistently contribute to the activation time regardless of AV nodal conditions. Combining these in programming ensures that both the intrinsic activation of the septum and RV consistently contribute to the QRS in the activation sequence. This technique differs from direct pacing of the computed tomography septum, which, to date, has not shown any true benefit—but, additionally, septal positions when analyzed by CT scan may not truly be on the septum.^27,28^

As most techniques such as Q-LV and programmer-based algorithms use RV-sense and RV-paced to LV times, whereas CRT is initiated LV to RV and epicardially, there exists built-in latency activating from the opposing direction of these measurements.^29,30^ One potential limitation of these methods may be as simple as not accounting for a unidirectional block or differences in the activation times from LV–RV times as previously reported.^31^ We programmed the longer of the LV–RV vectors as the LV-1 contributor in the final programming to ensure that the latest LV site began its activation while allowing time for the septum to also contribute. The faster LV–RV time programmed as the LV-2 contributor ensures as much time to activate the largest virtual LV electrode as possible while maintaining enough time for all the wavefronts from the septum, LV-1, LV-2, and the RV to activate with all programs being as close to simultaneous as possible.

As the ECG is the most widely available tool available in all stages of implanting and testing of CRT patients, it is probably the most used diagnostic constant in the continuum that a cardiac patient encounters in exchanges with their care providers. Furthermore, the QRSd is frequently assessed at implant in an attempt to maximize lead placement based on various conduction timing measurements such as qLV, paced RV–LV times, and V–V optimization.^18,28,32–38^ The benchmark of a positive initial deflection in lead V1 and axis deviation has been the hallmark of what we expect to see in the traditional ECGs of CRT patients.^34^ However, these were established and used prior to evolutions such as MPP, fusion attempts, LV-only pacing, and newer approaches to CRT optimization. As morphology is critical and most focus on this aspect of surface ECG optimization, no studies have compared the greatest V1 amplitude programming versus the narrowest QRSd post-CRT implantation with MPP in order to establish best clinical practices. Additionally, the impact of QRS shortening with MPP or the addition of septal fusion and their long-term clinical outcomes have also remained unassessed.

**Figure 4:**
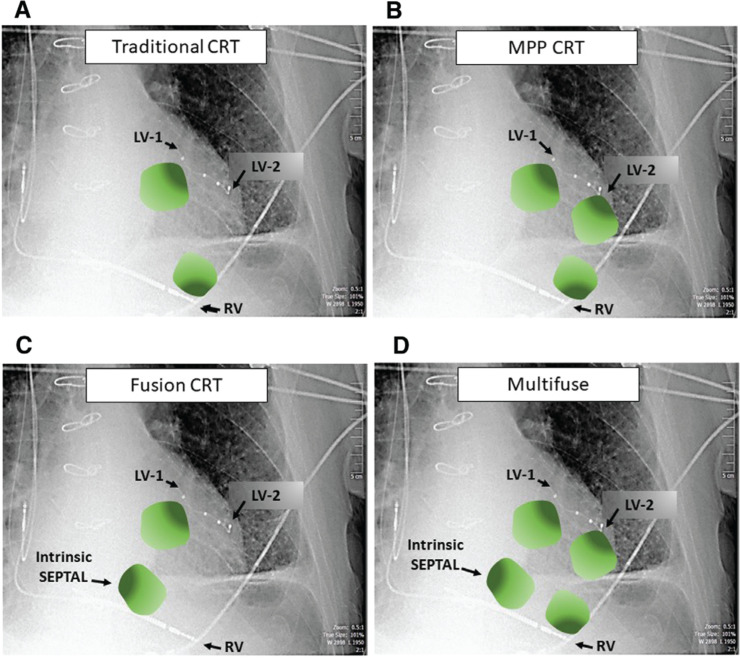
**A:** Traditional CRT with impulses originating from a single site on both the RV and LV leads. **B:** MPP with impulses originating from a single RV site and two LV activation sites. MPP: multipoint pacing. **C:** Fusion CRT where intrinsic septal activation occurs with a single activation site from the LV lead. CRT: cardiac resynchronization therapy; LV: left ventricular; RV: right ventricular. **D:** Combined impuses originating from two RV sites (septal activation and RV pacing) and two LV activation sites.

Throughout a CRT patient’s clinical encounters, programming, lead placement, and timing optimization are all techniques used to fuse multiple wavefronts and to provide narrowing of the QRSd in hopes of restoring mechanical synchronization. We historically attempted this using pacing leads placed into the RV and LV by way of the coronary sinus. This produces two wavefronts to create better electromechanical synchrony. The addition of MPP adds yet another third wavefront in CRT therapy. Our novel technique introduces the proposition that, with one more fourth optimized wavefront of consistent septal fusion in CRT–MPP patients, there appears initially to be an added benefit with regard to narrowing of the QRS duration **(Figure 4)**.

### Limitations

This method of fusion demonstrated significant reductions in the post-CRT implantation QRSd. As this study was limited in size to a small series of consecutive patients, a trial with a larger sample size would better confirm these findings. A randomized trial considering both QRSd narrowing and hard outcomes data including echocardiographic validation of response measures such as ejection fraction and end-systolic volume should be considered to better demonstrate any long-term clinical response. Further analysis of the incremental clinical benefit of each of these steps should also be weighed against the potential sequelae such as the impact on device longevity and compared against their added value.

## Conclusions

MFP demonstrated statistically significant reductions in QRSd in all patients tested in this analysis. The benefit was also significantly better in the sLBBB group as compared with in the IVCD group. This easy-to-calculate formula can be applied in all stages of CRT, provides dynamic optimization without the use of additional equipment, and can be performed in just minutes. Further outcomes-focused studies should be performed to validate our findings.

## Figures and Tables

**Table 1:** Patient Demographics

	All Patients	sLBBB Group	IVCD Group	p-value
Patients, n (%)	22	7	15	
Male sex, n (%)	19 (86.4%)	6 (85.7%)	13 (86.6%)	N/S
Age, years	66.6 ± 9.71	66.1 ± 8.0	67.9 ± 13.3	N/S
BMI, kg/m^2^	34.05 ± 10.4	32.9 ± 5.2	36.4 ± 17.6	N/S
CABG, n (%)	8/22 (34.8%)	2/7 (28.5%)	6/15 (40%)	N/S
HTN, n (%)	17/22 (73.9%)	5/7 (71.4%)	11/15 (73.3%)	N/S
COPD, n (%)	8/22 (34.8%)	2/7 (28.6%)	6/15 (40%)	N/S
Baseline QRSd, ms	153.3 ± 24.6	168.85 ± 27.29	146.06 ± 20.29	0.039
SRAT, ms	59.4 ± 28.49	47.14 ± 28.85	65.1 ± 27.3	N/S
SyncAV offset, ms	20.0 ± 24.97	17.14 ± 21.38	21.3 ± 27.1	N/S
Longest LV–RV time, ms	133.69 ± 61.06	116 ± 70.32	139 ± 62.08	N/S
Final QRSd, ms	115.31 ± 16.31	113 ± 16.69	116 ± 16.66	
QRSd reduction, ms	37.99	55.42 ± 19.3	30.07 ± 16.41	0.00046
QRSd, %	24.79%	32.1%	20.58%	N/S

All values reported as mean ± standard deviation or percent (%).

BMI: body mass index; CABG: coronary artery bypass surgery; COPD: chronic obstructive pulmonary disease; HTN: hypertension; IVCD: intra-ventricular conduction delay; LV: left ventricular; QRSd: QRS duration; RV: right ventricular; sLBBB: strictly defined left bundle branch block; SRAT: septal onset to right ventricular time.

**Table 2:** QRS Duration Changes

	Baseline	MFP	Reduction, ms (%)	p-value*
All	153 ± 24.6	115.31 ± 16.31	37.99 ± 21.07 (75.21%)	< 0.0001
sLBBB	168.85 ± 27.29	113 ± 16.69	55.42 ± 19.30 (32.1%)	0.0003
IVCD	146.06 ± 20.29	116 ± 16.66	30.07 ± 16.41 (20.6%)	0.0001

IVCD: intra-ventricular conduction delay; MFP: multifuse pacing; QRSd: QRS duration; sLBBB: strictly defined left bundle branch block.

*When comparing reduction in ms of QRSd.
